# Seasonal Variation of Newly Notified Pulmonary Tuberculosis Cases from 2004 to 2013 in Wuhan, China

**DOI:** 10.1371/journal.pone.0108369

**Published:** 2014-10-10

**Authors:** Xiaobing Yang, Qionghong Duan, Jianjie Wang, Zhengbin Zhang, Gaofeng Jiang

**Affiliations:** 1 Department of Tuberculosis Control, Wuhan Tuberculosis Institution, Wuhan, Hubei, China; 2 Department of Infectious Diseases Prevention and Control, Wuhan Center for Disease Prevention and Control, Wuhan, Hubei, China; 3 School of Public Health, Medical College, Wuhan University of Science and Technology, Wuhan, Hubei, China; St. Petersburg Pasteur Institute, Russian Federation

## Abstract

**Background:**

Although there was a report about the seasonal variation in Wuhan city, it only analyzed the prevalence data of pulmonary tuberculosis (TB) cases, and just studied the seasonality by subgroup of smear positive and negative from 2006 to 2010 by spectral analysis. In this study, we investigated the seasonality of the total newly notified pulmonary TB cases by subgroups such as time period, sex, age, occupation, district, and sputum smear result from 2004 to 2013 in Wuhan by a popular seasonal adjustment model (TRAMO-SEATS).

**Methods:**

Monthly pulmonary TB cases from 2004 to 2013 in Wuhan were analyzed by the TRAMO-SEATS seasonal adjustment program. Seasonal amplitude was calculated and compared within the subgroups.

**Results:**

From 2004 to 2013, there were 77.76 thousand newly notified pulmonary TB cases in Wuhan, China. There was a dominant peak spring peak (March) with seasonal amplitude of 56.81% and a second summer peak (September) of 43.40%, compared with the trough month (December). The spring seasonal amplitude in 2004–2008 was higher than that of 2009–2013(P<0.05). There were no statistical differences for spring seasonal amplitude within subgroups of gender, age, district, and sputum smear result (P>0.05). However, there were significant differences in spring seasonal amplitude by occupation, with amplitude ranging from 59.37% to 113.22% (P<0.05). The summer seasonal amplitude in 2004–2008 was higher than that of 2009–2013(P<0.05). There were no statistical differences in summer seasonal amplitude within subgroups of gender, district, sputum smear result(P>0.05). There were significant differences in summer seasonal amplitude by age, with amplitude ranging from 36.05% to 100.09% (P<0.05). Also, there were significant differences in summer seasonal amplitude by occupation, with amplitude ranging from 43.40% to 109.88% (P<0.05).

**Conclusions:**

There was an apparent seasonal variation in pulmonary TB cases in Wuhan. We speculated that spring peak in our study was most likely caused by the increased reactivation of the latent TB due to vitamin D deficiency and high PM2.5 concentration, while the summer peak was mainly resulted from the enhanced winter transmission due to indoor crowding in winter, overcrowding of public transportation over the period of the Spring Festival and health care seeking delay in winter.

## Introduction

Although China has achieved a great progress in TB control, tuberculosis is still a public health problem [1]. In 2012, it was estimated that there were approximately 1.0 million newly diagnosed cases, 1.4 million prevalent cases, and 44 thousand deaths that were due to TB in China [2].

In 1996, Douglas reported an unique seasonal pattern (summer peak) of tuberculosis compared with most other respiratory diseases [3]. Since then, there were many other researchers recognized various patterns of seasonality of TB [4]–[15], except for a few studies in which no obvious pattern of seasonality of TB was found [16]. Some studies showed that there was a single spring-early summer peak [4]–[8], while other studies found a single summer peak [3], [9], [10]. Additionally, a few of other studies showed a dominant peak (spring/summer) along with a second peak (summer/winter) [11], [12]. What caused the difference?

Researchers linked the seasonal variation of TB to two kinds of factors. Firstly, factors like indoor crowding in winter could lead to an increase of TB transmission (extrinsic infection). Secondly, vitamin D deficiency and high epidemic of other respiratory diseases could cause impaired immunity which would result in TB reactivation (intrinsic reactivation). However, questions arise. Which factor is the dominant reason for seasonality of TB? Are these the only influencing factors in term of extrinsic infection or intrinsic reactivation of TB? To examine those issues, we studied the seasonality of TB in Wuhan, China. Wuhan city with 10 million residents is the fourth largest city in China, and also one of the most important transportation junctions for the whole country.

There are only a few studies reporting the seasonal variation of TB in China till now. The first one was conducted in Hong Kong from 1991 to 2002 [10], the second one was about the whole country from 2004 to 2012 [15], and the third study explored the seasonality of pulmonary TB in Wuhan from 2006 to 2010 [12]. But the above report in Wuhan mentioned prevalence data of pulmonary TB cases, and studied the seasonality only by subgroup of smear positive and negative. In this study, we used newly notified pulmonary TB data, and investigated the seasonality of total pulmonary TB cases and by subgroups such as time period, sex, age, occupation, district, and sputum smear result from 2004 to 2013 in Wuhan.

Although there are a lot of mathematic models used to analyze the seasonality, TRAMO-SEATS (Time Series Regression with ARIMA Noise, Missing Observations and Outliers, TRAMO-SEATS) applied in our study is one of the most popular seasonal adjustment time series models over the world. Our study explored the seasonal variation of pulmonary TB in Wuhan, aimed to give the clue to reveal the characteristics of TB epidemic and the influencing factors and to develop suitable TB control measurements.

## Materials and Methods

### Data

The Data of confirmed pulmonary TB cases (laboratory or clinical verified) by month in Wuhan city, Hubei Province, China from Jan 2004 to Dec 2013 was obtained from the internet-based National Infectious Diseases Reporting System(NIDRS), Chinese Center for Disease Control and Prevention. It is mandatory for all hospitals, clinics, disease prevention and control institutions and other designated health care establishments to report all newly diagnosed pulmonary TB cases timely and directly via NIDRS. From NIDRS, we also obtained the data of sex, age, occupation, district, and sputum smear result of pulmonary TB cases.

### Seasonal Adjustment of Time Series

Monthly pulmonary TB case counts were analyzed by the TRAMO-SEATS seasonal adjustment program which is an advanced seasonal adjustment program paralleled with X-12-ARIMA program [17]. It is a very stable and efficient model developed by the bank of Spain. In the TRAMO-SEATS program, the original time series is decomposed into three basic components: trend component, seasonal factor and irregular noise component. The trend component is the long-term and medium-to-long term variation tendency of the time series, including up or down trend and important break points; it reflects the basic and primary trend of a disease in the long-term or medium-to-long term. The seasonal factor is considered as a fluctuation repeatedly occurred in the same month or quarter each year; it shows the seasonal characteristic of the disease, and caused by complex factors. The irregular noise component is the residual component that remains after trend component and seasonal factor are removed from the time series; it represents the noise of the time series.

### Statistical Analysis

Firstly, the TRAMO-SEATS program was applied to the raw monthly case counts. The time series of total pulmonary TB cases was decomposed into trend component, seasonal and irregular noise component. A decomposition of monthly case counts was obtained for groups of interest, according to time period, sex, age, occupation, district, and sputum smear result. If the population had the identifiable seasonality judged by TRAMO-SEATS program, we calculated the mean spring peak month, mean summer peak month, mean trough month, annual spring seasonal amplitude and annual summer seasonal amplitude with 95% confidence interval (CI), and annual spring-summer amplitude difference with median, upper and lower quartile for the years from 2004 to 2013. Annual spring and summer seasonal amplitude were calculated from isolated seasonal factor and defined as the fraction with the numerator being the spring-peak-to-trough or summer-peak-to-trough difference between the months with the highest spring or summer and the lowest case counts and with the denominator being mean case counts for that year. Annual spring-summer amplitude difference was defined as a fraction with the numerator being the spring-peak-to-summer-peak difference between the months with the highest spring and the highest summer case counts and with the denominator being mean case counts for that year.

Secondly, the amplitudes of spring and summer seasonal fluctuation were compared within groups. The Student’s *t*-test for two independent samples was used to compare seasonal amplitudes of two subgroups. The Bonferroni method for one way analysis of variance was used to compare all pairwise seasonal amplitudes of three or more subgroups in condition of equal variance and normal distribution, and the Kruskal-Wallis test was used in condition of unequal variance or skew distribution. P-value<0.05 was considered statistically significant.

### Statistical Software

The DEMETRA package (DEMETRA 2.0; Eurostat, the Statistical Office of the European Communities) was used for TRAMO-SEATS program; Microsoft Excel 2007 (Microsoft Corporation, Redmond, WA, USA) was used for data management; the SPSS 18.0 (SPSS Inc., Chicago, IL, USA) was used for statistical comparisons of seasonal indicators within subgroups.

## Results

**Figure 1:**
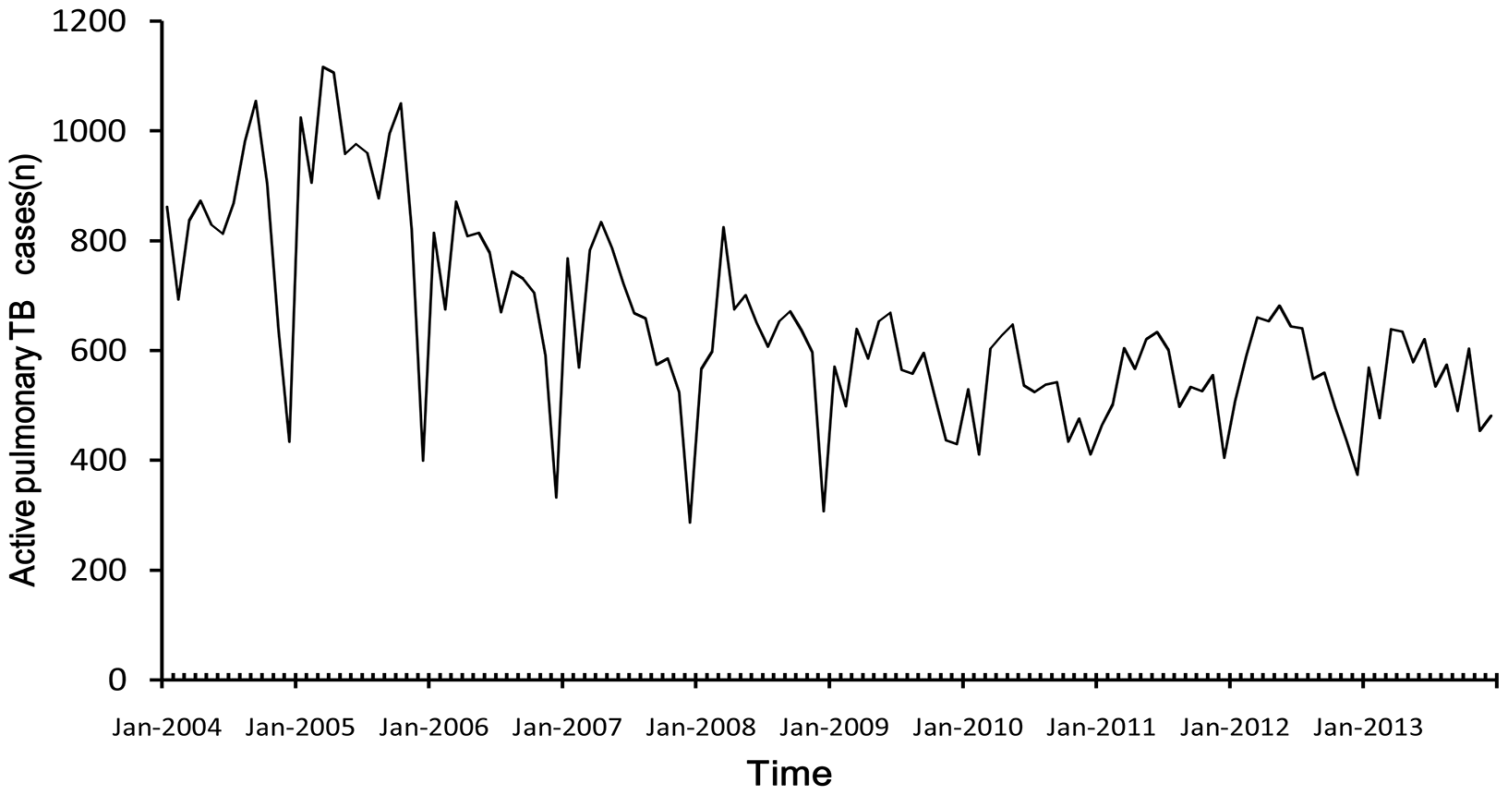
The original time series of pulmonary TB cases.

From 2004 to 2013, there were 77.76 thousand newly notified pulmonary TB cases in Wuhan, China (Table 1). Figure 1 showed the original time series of pulmonary TB cases from 2004 January to 2013 December. Wuhan city is located in Hubei Province which is in the middle of China. Wuhan has a subtropical wet monsoon climate. Based on the temperature data and climatic seasonal division method, seasons in Wuhan were defined as spring (March–May), summer (June–September), autumn (October–November), and winter (December–February) [18]. There were a dominant spring peak (March) and a second summer peak (September), along with the trough (December).

**Table 1:** Pulmonary TB cases in Wuhan, 2004–2013.

Group		2004	2005	2006	2007	2008	2009	2010	2011	2012	2013
All PTB cases		9788	11196	8541	7765	7493	6723	6285	6513	6797	6660
sex	Male	7068	8161	6094	5697	5496	4872	4528	4695	4942	4757
	Female	2720	3035	2447	2068	1997	1851	1757	1818	1855	1903
	0–14	100	106	79	48	44	50	35	32	41	40
Age(years)	15–24	1729	1839	1638	1462	1557	1456	1378	1407	1304	1239
	25–44	3063	3579	2730	2304	2165	1893	1764	1816	1889	1802
	45–64	3055	3587	2642	2497	2457	2222	2076	2184	2261	2344
	65+	1841	2085	1452	1454	1270	1102	1032	1074	1302	1235
Occupation	Peasant	3503	3632	2557	2124	1977	1771	1523	1479	1509	1288
	Migrant worker	237	231	145	139	157	119	128	125	88	23
	Worker	830	1074	798	767	617	503	514	322	229	187
	Student	983	1016	820	689	629	705	597	534	493	475
	Others	4235	5243	4221	4046	4113	3625	3523	4053	4478	4687
District	Center	4934	6080	4761	4489	4253	3625	3457	3622	3669	3711
	Far	4854	5116	3780	3276	3240	3098	2828	2891	3128	2949
Sputum smear	Positive TB	2724	4136	3217	3317	3128	3286	2958	3082	2844	2873
	Negative TB	4034	5054	4756	4341	4225	3392	3294	3411	3935	3776
	Unknown	3030	2006	568	107	140	45	33	20	18	11

**Figure 2:**
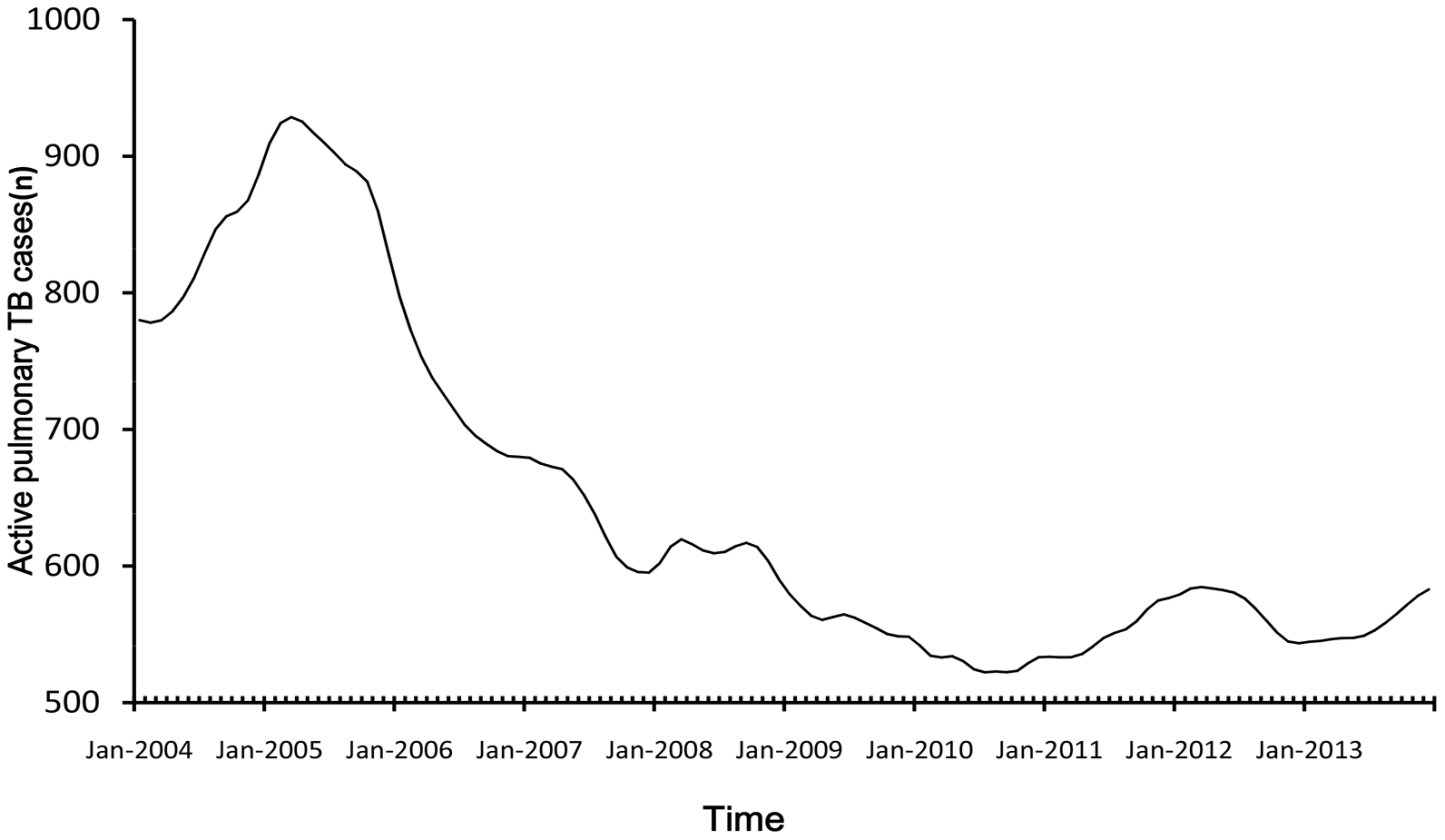
The trend component of time series.

**Figure 3:**
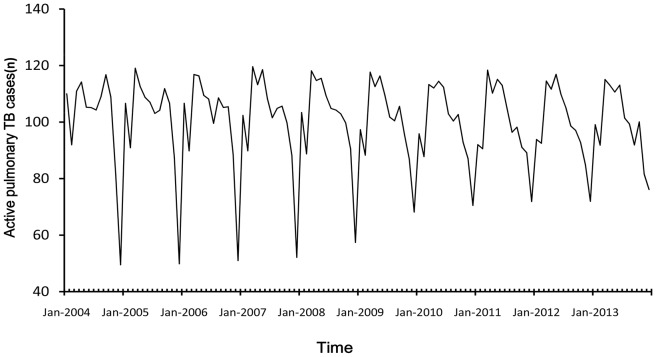
The seasonal component of time series.

**Figure 4:**
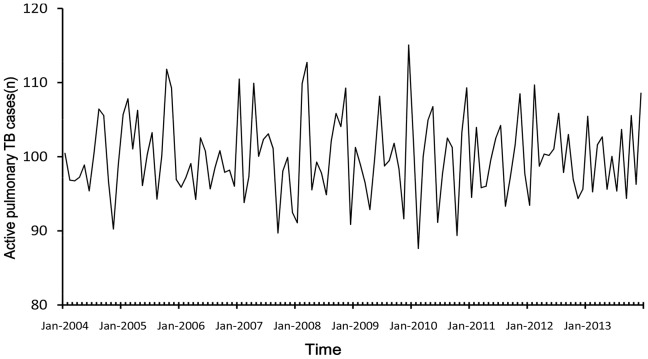
The irregular noise component of time series.

Figure 2, 3 and 4 displayed the three components produced by TRAMO-SEATS program which were trend component, seasonal and irregular noise component. There was an upward trend from 2004 to 2005, a decreasing trend from 2005 to 2010, then a slowing upward trend from 2010 to 2013. From the isolated seasonal component, it was found that seasonal amplitude decreased each year from 2004 to 2013.

Table 2 illustrated spring seasonal amplitudes for subgroups of each group and the comparisons of spring seasonal amplitudes. An annual mean of 56.81% (95% CI, 41.93%–71.69%) more pulmonary TB cases were notified in the spring peak month (March) compared with the trough month (December) from 2004 to 2013. The spring seasonal amplitude in 2004–2008(73.17%) was higher than that of 2009–2013(40.45%), and the difference between them was significant (P<0.05). There were no statistical differences in spring seasonal amplitude between males and females, age groups, center and far district, smear positive and smear negative pulmonary TB (P>0.05). There were significant differences in spring seasonal amplitude by occupation, with amplitude ranging from 59.37% to 113.22% (P<0.05). The most common of spring peak month was March, the others were April and May. The trough month for all groups and subgroups was December.

**Table 2:** Spring seasonal amplitude of pulmonary TB cases in Wuhan, 2004–2013.

Group		Spring peak/trough month	Seasonal amplitude(%)	SE(%)	95%CI(%) lower	95%CI(%) upper	*P* value
All PTB cases		Mar/Dec	56.81	6.29	41.93	71.69	
Period	Year 2004–2008	Mar/Dec	73.17	5.00	59.29	87.05	0.001§
	Year 2009–2013	Mar/Dec	40.45	4.40	28.24	52.66	
Sex	Male	Mar/Dec	58.01	6.18	43.39	72.63	0.831§
	Female	Mar/Dec	55.88	7.65	37.79	73.97	
Age(years)	0–14	Apr/Dec	91.05	21.73	39.66	142.44	0.081¶
	15–24	Mar/Dec	74.55	6.14	60.03	89.07	
	25–44	Apr/Dec	54.74	6.35	39.72	69.76	
	45–64	Mar/Dec	56.52	6.34	41.53	71.51	
	65+	May/Dec	66.64	10.52	41.76	91.52	
Occupation	Peasant	May/Dec	59.37	9.10	37.85	80.89	0.003¶
	Migrant worker	Apr/Dec	76.10	27.53	10.99	141.21	
	Worker	Mar/Dec	64.57	8.28	44.99	84.15	
	Student	Mar/Dec	113.22	11.39	86.28	140.16	
District	Center	Mar/Dec	54.76	6.10	40.33	69.19	0.702§
	Far	Mar/Dec	58.47	7.34	41.11	75.83	
Sputum smear	Positive TB	Mar/Dec	62.22	6.20	47.56	76.88	0.848§
	Negative TB	Mar/Dec	60.22	8.22	40.78	79.66	

Abbreviation:SE,standard error; CI,confidence interval.

§ Two-tailed two dependent samples Student's *t*-test for difference in seasonal amplitudes.

¶ Kruskal-Wallis test for all pairwise multiple comparison among seasonal amplitudes.

Table 3 summarized summer seasonal amplitudes for subgroups of each group and the comparisons of summer seasonal amplitudes. An annual mean of 43.40% (95% CI, 28.88%–57.92%) more pulmonary TB cases were notified in the summer peak month (September) compared with the trough month (December) from 2004 to 2013. The summer seasonal amplitude in 2004–2008(60.11%) was higher than that of 2009–2013(26.68%), and the difference between them was significant (P<0.05). There was no statistical difference in summer seasonal amplitude between males and females, center and far district, smear positive and smear negative pulmonary TB (P>0.05). There were significant differences in summer seasonal amplitude by age, with amplitude ranging from 36.05% to 100.09% (P<0.05). There were significant differences in summer seasonal amplitude by occupation, with amplitude ranging from 43.40% to 109.88% (P<0.05). The most common of summer peak month was September, others were July and August. The trough month for all groups and subgroups was December.

**Table 3:** Summer seasonal amplitude of pulmonary TB cases in Wuhan, 2004–2013.

Group		Summer peak/trough month	Seasonal amplitude (%)	SE(%)	95%CI(%) lower	95%CI(%) upper	*P* value
All PTB cases		Sep/Dec	43.40	6.14	28.88	57.92	
Period	Year 2004–2008	Sep/Dec	60.11	5.12	45.90	74.32	0.000§
	Year 2009–2013	Sep/Dec	26.68	1.99	21.16	32.20	
Sex	Male	Sep/Dec	42.19	7.08	25.45	58.93	0.389§
	Female	Sep/Dec	49.83	4.98	38.05	61.61	
Age(years)	0–14	July/Dec	100.09	14.91	64.83	135.35	0.003¶
	15–24	Sep/Dec	73.69	9.38	51.51	95.87	
	25–44	Sep/Dec	36.05	6.09	21.65	50.45	
	45–64	Aug/Dec	44.19	7.65	26.10	62.28	
	65+	Aug/Dec	57.61	9.49	35.17	80.05	
Occupation	Peasant	Aug/Dec	49.84	7.69	31.65	68.03	0.003¶
	Migrant worker	Sep/Dec	55.16	17.39	14.03	96.29	
	Worker	July/Dec	43.40	6.83	27.25	59.55	
	Student	Sep/Dec	109.88	15.91	72.25	147.51	
District	Center	Sep/Dec	44.05	8.24	24.56	63.54	0.545§
	Far	Aug/Dec	50.34	5.99	36.17	64.51	
Sputum smear	Positive TB	Sep/Dec	58.23	6.74	42.29	74.17	0.112§
	Negative TB	Sep/Dec	41.52	7.41	24.00	59.04	

Abbreviation:SE,standard error;CI,confidence interval.

§ Two-tailed two dependent samples Student's *t*-test for difference in seasonal amplitudes.

¶ Kruskal-Wallis test for all pairwise multiple comparison among seasonal amplitudes.

Table 4 displayed the median, the lower and upper quartile of spring and summer seasonal amplitude difference in Wuhan from 2004 to 2013. The median of 0–14 years old group and student were negative which showed that the summer seasonal amplitude was higher than the spring seasonal amplitude.

**Table 4:** The difference of spring and summer seasonal amplitude of pulmonary TB cases in Wuhan, 2004–2013.

Group		Spring peak/summer peak month	Seasonal amplitude difference(%)(median)	Upper quartile	Lower quartile	Interquartile range
All PTB cases		Mar/Sep	15.53	21.17	9.13	12.03
Period	Year 2004–2008	Mar/Sep	17.98	24.50	12.97	11.53
	Year 2009–2013	Mar/Sep	13.08	20.05	7.85	12.19
Sex	Male	Mar/Sep	17.84	25.21	14.17	11.04
	Female	Mar/Sep	5.66	12.99	0.17	12.82
Age(years)	0–14	Apr/July	−6.34	35.63	−44.32	79.94
	15–24	Mar/Sep	8.09	19.73	−18.45	38.18
	25–44	Apr/Sep	20.05	27.58	14.00	13.58
	45–64	Mar/Aug	13.05	24.39	6.40	17.99
	65+	Mar/Aug	5.38	22.58	0.41	22.17
Occupation	Peasant	Mar/Aug	13.58	21.03	7.13	13.89
	Migrant worker	Apr/Sep	9.84	38.04	−16.92	54.96
	Worker	Mar/July	19.45	44.87	9.32	35.55
	Student	Mar/Sep	−2.90	32.64	−27.45	60.09
District	Center	Mar/Sep	14.30	25.46	4.31	21.15
	Far	Mar/Aug	7.09	19.50	−2.84	22.33
Sputum smear	Positive TB	Mar/Sep	7.64	14.93	−8.98	23.90
	Negative TB	Mar/Sep	16.39	21.87	7.79	14.07

## Discussion

After the severe acute respiratory syndrome (SARS) outbroke in 2003, the internet-based National Infectious Diseases Reporting System (NIDRS) was established in China. The government has taken a series of measures to strengthen the mandatory reporting system of infectious diseases [19]. From then on, the hospitals have taken it as a serious task to report the legal infectious diseases. Thus, the surveillance and reporting system of legal infectious diseases become more reliable than before [19]. In China, pulmonary tuberculosis is one of the legal infectious diseases.

A procedure that filters the seasonal fluctuations from a time series is called a seasonal adjustment program. There exists a lot of different procedures but most of the statistical agencies use quite standardized techniques where the most important ones are the X-12-ARIMA program and the TRAMO/SEATS procedure [20]. Comparing with the X-12-ARIMA, TRAMO/SEATS has the characteristics of high stability and efficiency [17].

TRAMO does the pre-adjustment of the series and stands for “Time Series Regression with ARIMA Noise, Missing Observations and Outliers”. It does a similar job as regARIMA in the X12 program. SEATS stands for “Signal Extraction in ARIMA Time Series”, and decomposes the series in its unobserved components following an ARIMA based method [20]. The TRAMO and SEATS packages were developed at the Bank of Spain by Victor Gomez and Agustin Maravall, with the programming help of Gianluca Caporello. Eurostat released the program package DEMETRA, which included X12 and the TRAMO/SEATS programs [17].

We found that there was an apparent seasonal variation of pulmonary TB disease in Wuhan city in our study. The dominant (spring) peak month was March for pulmonary TB and the trough month was December in this study. The spring peak month was similar to many of the studies in other locations such as Japan [4], Kuwait [5], India [6] and Spain [11]. The trough month was earlier than that (January or February) in Japan [4], Hong Kong [10] and Hubei Province [15], while was same as the research in Spain [11], Mongolia [8], India [6] and Kuwait [5]. In our study, the second (summer) peak existed which was consistent with studies conducted in Hong Kong [10] and United Kingdom [3]. The summer peak month was September for the total pulmonary TB cases and the majority of subgroups.

A number of factors enhance the pulmonary TB transmission in winter. First, poor ventilated room crowded with people could increase the chance of transmission among the infectious source and the contactors in winter. In Wuhan city, the winter is from December to February, and the coldest month is January in most of the years. In winter, people are more likely to stay at home and close the window because of the low temperature, moreover, because of the severe air pollution.

Second, the crowding and poor ventilated public transport plays an important role in the transmission of pulmonary TB [21]–[23]. The most important holiday (Spring Festival, Chinese New Year) in China is always at the end of January or at the beginning of February. Transportation during the Spring Festival holiday, called “chunyun” in china, is very busy. During the period of about half month before or after the Spring Festival, there are thousands of people taking buses/coaches, airplanes or railways to travel between the hometown and the work town. The crowding degree of the above public transports is several times as the usual time. Furthermore, the travel time of people on these public transports is longer than usual, pulmonary TB has more chance to transmit over “chunyun” time period.

Third, the severe air pollution especially haze could increase TB transmission. The first time that Wuhan residents obviously felt haze weather which caused peripheral visibility extremely reduced is June 12, 2012. From then on, haze weather occurred frequently. Many studies showed that, the main component of haze was inhalable airborne particle matter (mainly PM 2.5) [24], [25]. From January 1, 2013, Wuhan began to release hourly PM2.5 readings. PM2.5 concentration is highest in winter. Although the main components of PM2.5 are chemicals such as SO_4_^2−^, NO_3_^−^ and NH_4_^+^, PM2.5 still has the possibility to contain Mycobacterium tuberculosis [26], [27], thus increases the TB transmission.

Fourth, health care seeking delay in winter could increase the risk of disease transmission. The weather in winter is moderately cold in Wuhan. The cold weather may be a possible cause leading to health care seeking delay in winter. Another important reason for health care seeking delay may be the Spring Festival at the end of winter. During the Spring Festival, congregation for celebrating the coming new year always leads to heath care seeking delay. Thus, the consultation rate during this period is relatively low. This delay in health care seeking contributes to diagnosis delay which may increase the risk of disease transmission (due to the longer communicable period of tuberculosis) in winter.

The nearly recent molecular epidemiological study revealed that the median incubation time of TB was about 15.6 months [28]. Moreover, from the onset of symptoms to the diagnosis of TB, there is a time interval which is called diagnosis delay. The median diagnosis delay of pulmonary TB cases in Wuhan was 35 days in our previous study [29]. After the increase of TB transmission in winter, the peak of TB cases would appear 16.8 months (the sum of incubation and diagnosis delay) later. In other words, the summer peak (July-September) in this study was most likely resulted from the winter transmission. In our study, the 0–14 years old age group had the highest summer amplitude which paralleled with the spring amplitude. The summer amplitude of 0–14 years old age group was significant higher than older age groups. The younger persons must have had relatively recent TB infection in winter compared with the elderly who may have been infected many years earlier. This suggested a possible organic basis for the above speculation.

The reactivation of TB means that the relatively longer time between the acquisition of mycobacterium tuberculosis and the onset of the symptoms comparing with the winter transmission. The reactivation of tuberculosis is associated with HIV infection, immunosuppressive therapy or complex factors such as poor nutrition and socioeconomic status which can lead to decreased immunity. First, vitamin D deficiency has been hypothesized to be contributed to the reactivation of TB [30]. Prior to the introduction of chemotherapy, the measures (sunlight exposure and fish liver oils supplement) to enhance the level of serum 25-hydroxy vitamin D (25(OH)D) were included as an integral part of traditional anti-TB therapy. Studies [31], [32] showed that vitamin D deficiency could impair cell mediated immunity and reduce function of macrophages, thus lead to the reactivation of tuberculosis. Synthesis of vitamin D is dependent on the sunlight. Latitude is considered as an important influencing factor of sunlight exposure. The consensus reached in nutrition is that, the serum 25 (OH) D concentration is quite low or absent above latitude of 33° N in winter [33]. Although there has been no data of the seasonal variation of serum 25 (OH) D concentrations of residents in Wuhan which is located at 30° N, we suppose that the serum 25 (OH) D concentrations are relatively low in winter of Wuhan residents. The result of one study showed that the serum level of 25-hydroxy vitamin D revealed about one month lag after the change of ultraviolet radiation [34], another study showed that the trough of 25-hydroxy vitamin D was in mid-winter [35]. Thus, the trough month of the serum level of 25-hydroxy vitamin D in Wuhan residents is assumed to be in February, one month behind the trough of ultraviolet radiation (January). This would result in the reactivation peak in late winter, considering the diagnosis delay (35 days, about 1.2 month), then would lead to the spring peak (March) of pulmonary TB.

Second, we speculate that ambient air pollution is related to TB reactivation. Several studies showed that after a time of exposure to diesel exhaust inhalation, the expression levels of interleukin (TNF)-α, IL-12, interferon (IFN)-γ in marine decreased significantly [36], [37]. IFN-γ attacks and kills the Mycobacterium tuberculosis by macrophage activation. TNF-α is important in the procedure of granuloma formation and the infection localized. Furthermore, the inhibition of TNF-α by drugs would lead to TB reactivation [38]. A recent study suggested a potential association between the exposure to particulate matter (PM) and pulmonary TB [39]. Additionally, the frequent haze weather may lead to reduced ultraviolet radiation intensity, thus lower the level of serum 25-hydroxy vitamin D in body. The haze weather is most frequently in winter. Consequently, all of the above factors would cause the winter or early spring peak of TB reactivation. Considering the diagnosis delay of TB, TB reactivation would manifest as TB peak in spring. Thus, we speculated that spring peak (from March to May) in our study was more likely caused by the reactivation of the latent TB than the enhanced winter transmission.

There are paucity of data on the relationship between the humidity, precipitation and seasonality of tuberculosis. Ane-Anyangwe's study [13] in the South Western Cameroon showed that the peak month was in winter (rainy season). The author attributed it to the two factors: vitamin D deficiency due to the lack of sunlight exposure, higher risks of indoor infection because of the humid and cold weather. The rainy season in Wuhan is very short, last about 20–30 days every year, usually from mid June to early July in summer. The precipitation does not have significant seasonality beyond the rainy season. The ultraviolet radiation is enough for the synthesis of serum 25(OH) D even during the rainfall season period. In rainy season, the temperature is high and the continuous heavy rain is infrequent(the annual mean frequency of continuous heavy rain was 0.41 times between 1959–1996, 95% lasted less than 5 days) [40], thus it is not likely to enhance the indoor infection.

There was no significant difference between the males and females in the spring and summer seasonal amplitude. This observation was similar to many of the studies on the seasonality of TB over the world [10], [41]. Even through the summer seasonal amplitude of smear positive was higher than that of smear negative pulmonary TB, which was similar to Luo's [12] and Leung's [10] study, but the difference between them was not significant.

The spring and summer seasonal amplitude of pulmonary TB over period 2009–2013 were significantly lower than that over 2004–2008. The seasonal variation reduced along with the number of pulmonary TB cases. The decrease of TB cases might be one of the reasons of the change of seasonal variation. The other reason might be the variation of the influencing factors driving the seasonality of TB, such as the increasing of ground public transport. The first section of the Wuhan subway system was opened on December 12, 2012, and more and more subways begin work now. The crowding and close environmental of subway cars would provide enough chance to the TB transmission.

Newly notification pulmonary TB cases had a decreasing trend from 2005 to 2010, then a slowing upward trend from 2010 to 2013. This suggested that during the recent period, the risk factors for transmission and reactivation of tuberculosis increased, and should arouse the attention of the government. In our opinion, these factors included the increasing of ground public transport, the more severe of air pollution, and so on.

Given the consistent pattern in seasonality of TB, foreign travelers can be informed about TB risk and screening. The consistency in seasonality of TB case detection may be used to initiate control measures and provide extra facilities and arrangements during peak seasons.

Finally, this study had some limitations. First, Lack of clinical data was a major limitation of this study. Except for the sputum smear result of the pulmonary TB cases, there were no data on the other clinical characteristics such as culture result, cavity, retreatment or new case, and so on in National Infectious Diseases Reporting System (NIDRS). As a result, those clinical factors that had been mentioned above were not included in the analysis. Second, all of the explanation of the seasonality was just the speculation, and the conclusions were not definite. Since the study is observational, the temporal association between cause and effect could not be established. There was a need of further study to explore the exact mechanism of the seasonal variation of pulmonary TB in Wuhan. The further research should be focused on the relationship between PM2.5 and serum 25 (OH) D concentration, PM2.5 and pulmonary TB, and so on. Thus the results would provide basis to prevent and control the TB epidemic.
